# CNN-based survival model for pancreatic ductal adenocarcinoma in medical imaging

**DOI:** 10.1186/s12880-020-0418-1

**Published:** 2020-02-03

**Authors:** Yucheng Zhang, Edrise M. Lobo-Mueller, Paul Karanicolas, Steven Gallinger, Masoom A. Haider, Farzad Khalvati

**Affiliations:** 1 Institute of Medical Science, University of Toronto, Toronto, ON Canada; 2 Lunenfeld-Tanenbaum Research Institute, Sinai Health System, Toronto, ON Canada; 3 Department of Radiology, McMaster University and Hamilton Health Sciences, Juravinski Hospital and Cancer Centre, Hamilton, Ontario Canada; 4 Department of Surgery, Sunnybrook Health Sciences Centre, Toronto, ON Canada; 5 Department of Medical Imaging, University of Toronto, Toronto, ON Canada; 6 Department of Mechanical and Industrial Engineering, University of Toronto, Toronto, ON Canada

**Keywords:** Cox proportional hazard model, Radiomics, Convolutional neural network, Survial analysis

## Abstract

**Background:**

Cox proportional hazard model (CPH) is commonly used in clinical research for survival analysis. In quantitative medical imaging (radiomics) studies, CPH plays an important role in feature reduction and modeling. However, the underlying linear assumption of CPH model limits the prognostic performance. In this work, using transfer learning, a convolutional neural network (CNN) based survival model was built and tested on preoperative CT images of resectable Pancreatic Ductal Adenocarcinoma (PDAC) patients.

**Results:**

The proposed CNN-based survival model outperformed the traditional CPH-based radiomics approach in terms of concordance index and index of prediction accuracy, providing a better fit for patients’ survival patterns.

**Conclusions:**

The proposed CNN-based survival model outperforms CPH-based radiomics pipeline in PDAC prognosis. This approach offers a better fit for survival patterns based on CT images and overcomes the limitations of conventional survival models.

## Background

In clinical practice, medical imaging plays an increasingly important role in informed decision making of clinicians for disease management. Radiomics is a systematic approach to study the latent information in medical imaging for improved accuracy in prognosis. A typical radiomics study involves image acquisition, feature extraction, feature analysis, and predictive modeling for a clinical outcome such as patient survival [1]. Efforts have been made to standardize quantitative imaging features (radiomic features) by implementing open source libraries such as PyRadiomics [2]. These feature banks contain thousands of hand-crafted formulas, designed to extract the distribution or texture information from medical images. In radiomics studies, a feature reduction method (e.g., principle component analysis) is used to select representative features [3]. The prognostic features are usually determined using Cox proportional hazard model (CPH) [4]. In the past decade, several radiomics features have shown prognostic value in different diseases especially different types of cancer [5–9]. However, the high dimensionality nature of radiomics features makes the feature selection prone to multiple testing, leading to false positives and low performance in the validation cohorts.

As a statistical method, survival models are commonly used in clinical research to identify potential risk factors and predict risks for a variety of clinical outcomes including patients’ overall survivals for different diseases such as cancer. CPH is one of the most commonly used survival analysis tools [10–12]. CPH is a type of semiparametric model that calculates the effects of features (independent variables) on the risk of a certain event (e.g., death) [13]. For example, CPH can measure the effect of tumor size on the risk of death.

The CPH-based survival models can help clinicians make more personalized treatment decisions for individual patients. Traditional CPH models assume that the independent variables make a linear contribution to the model, with respect to time [13]. In many conditions, this assumption oversimplifies the relationships between biomarkers (e.g., radiomic features) and outcomes, especially in cancer diseases with poor prognosis including Pancreatic Ductal Adenocarcinoma (PDAC) [11]. With a limited sample size, the violation of linear assumption may not be obvious. However, as data sizes increase, the violation of linear assumption in CPH models increasingly becomes more obvious and problematic, diminishing the performance and reliability of such models [10–12]. In modern survival modeling approaches, restricted cubic splines have been applied to fix this weakness of CPH models [14, 15]. However, most radiomics studies have failed to address this shortcoming and instead, either they have adopted binary classification methods discarding duration (time to event) information altogether or continued to use conventional CPH Methods [16–21].

The binary classification methods solve the nonlinearity by using a classifier such as Random Forest or Support Vector Machine (SVM) [22, 23]. Although these classifiers perform well in diagnosis and prognosis, they discard the time information in the modeling. For disease with poor prognosis such as pancreatic cancer, the 5-year survival rate is very low (e.g., less than 10% for pancreatic cancer) [24–26]. Consequently, binary predictions only offer limited information for clinicians in designing personalized treatment plans and hence, a nonlinear survival model that takes duration (time to an event such as death) into account to provide useful information on the survival is desired.

A recent development in artificial neural networks (ANNs) has provided an alternative solution for survival modeling. ANNs can learn complex and nonlinear relationships between prognostic features and an individual’s risk for a given outcome [27]. Therefore, the ANNs-based model can provide an improved personalized recommendation based on the computed risk. Nevertheless, previous studies have demonstrated mixed performance for risk-prediction models [27–29]. This may be due to the small sample size and limited feature space leading to ANNs models that are underfitted [28]. To exploit the ANNs architecture and successfully apply them to complex cases, larger datasets are required. Recent work has shown that, given enough sample sizes, ANNs can, in fact, outperform traditional CPH survival models [10–12].

The majority of previous works on deep learning based survival analysis including DeepSurv and NNET-survival are ANNs-based survival models with modified loss function to capture more accurate survival patterns [10, 11]. These models take features (e.g., age, gender, height) as input and return risks for patients at different timepoints. However, feeding radiomics features into these ANNs as input is not the optimal solution due to the multicollinearity issue.

In this research, we used medical images as input, replacing radiomics feature extractors with a Convolutional Neural Network (CNN) architecture to extract disease-specific image features which are associated with survival patterns. As the most well-known architecture in deep learning, CNNs extract imaging features by applying multiple layers of convolution operations to the images. Furthermore, the weights of the convolution filters are finetuned during training via backpropagation process [30, 31]. Thus, given sufficient data, CNNs can be used to extract disease-specific features, which can be used for diagnosis or prognosis purposes [32–35]. Although traditional medical imaging based CNNs use “binary” or “multinomial” classification loss function, the loss function can be modified to also capture the survival patterns [11]. By doing so, CNN can be tuned to extract features that are associated with the risk of the outcome in a certain duration. We hypothesized that the proposed CNN-based Survival (CNN-Survival) model with a modified loss function would outperform conventional radiomics and CPH-based prognosis models.

## Methods

### Data

Three independent cohorts were used in this study. Cohort 1 consists of publicly available pretreatment CT scans of 422 Non-small cell lung cancer (NSCLC) patients [7]. Cohort 2 has 68 resectable pancreatic adenocarcinoma (PDAC) patients collected from a local hospital from 2008 to 2013. Cohort 3, which is the test data, consists of 30 resectable PDAC patients enrolled in another independent hospital site from 2007 to 2012 [3]. For all the patients in these three independent cohorts, CT scans, annotations (contours) of tumor performed by radiologists, and survival data were available. For PDAC patients, the CT scans were preoperative contrast-enhanced images of resectable patients, and the survival data was collected from the date of surgery until death. CT images from all three cohorts were read from DICOM file without further processing. As CT scans were from different institutions, the image acquisition protocol information (e.g., exact contrast bolus volume, timing, and injection rate) was not consistent over the time period. The institutions’ Research Ethics Boards approved these retrospective studies and all methods were carried out in accordance with relevant guidelines and regulations.

### Architecture of the proposed CNN-survival

**Fig. 1:**
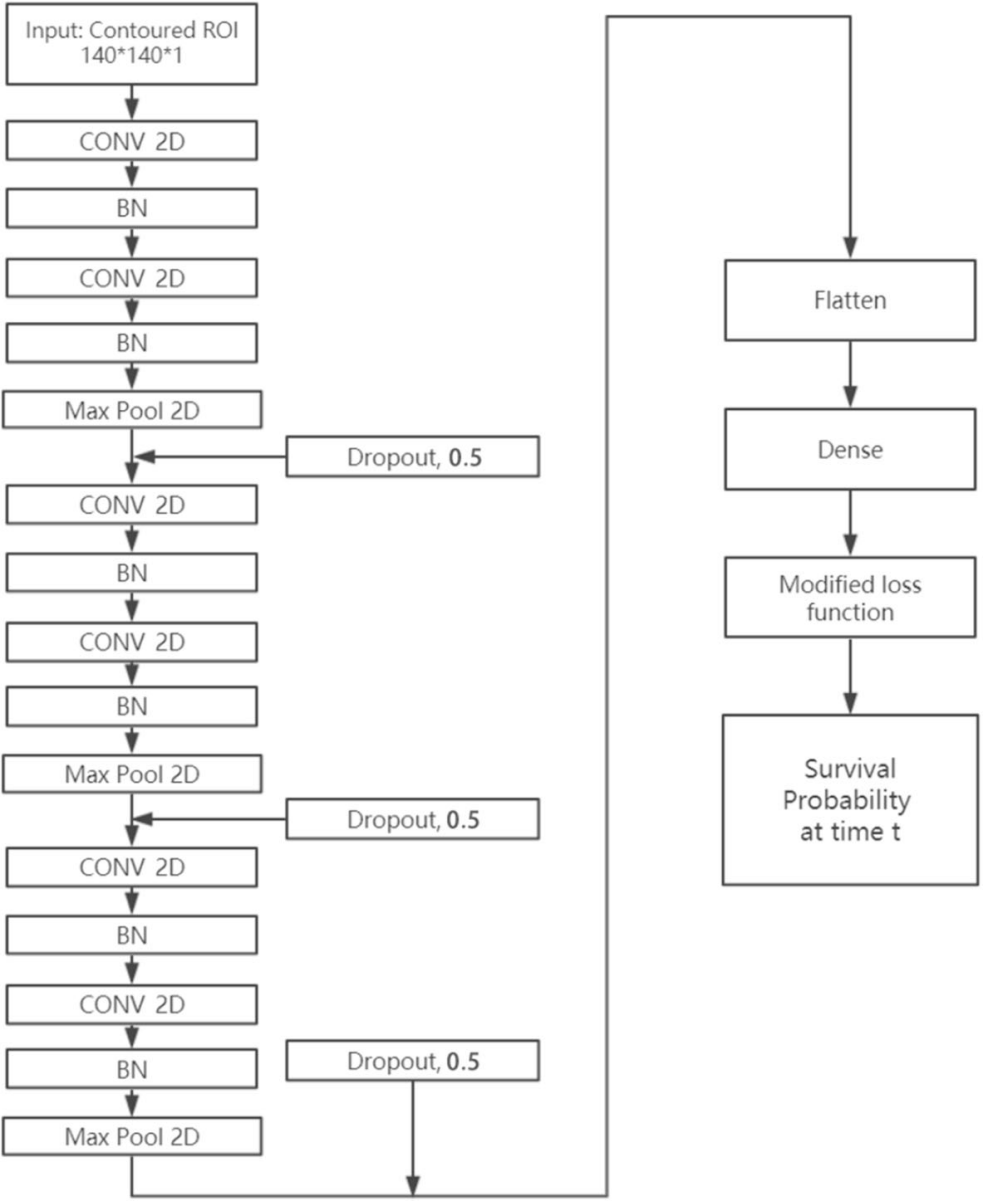
The proposed CNN-Survival architecture: 6-layer CNN, batch normalization, and Max Pooling layers. There are also three dropout layers to control the potential overfitting

**Fig. 2:**
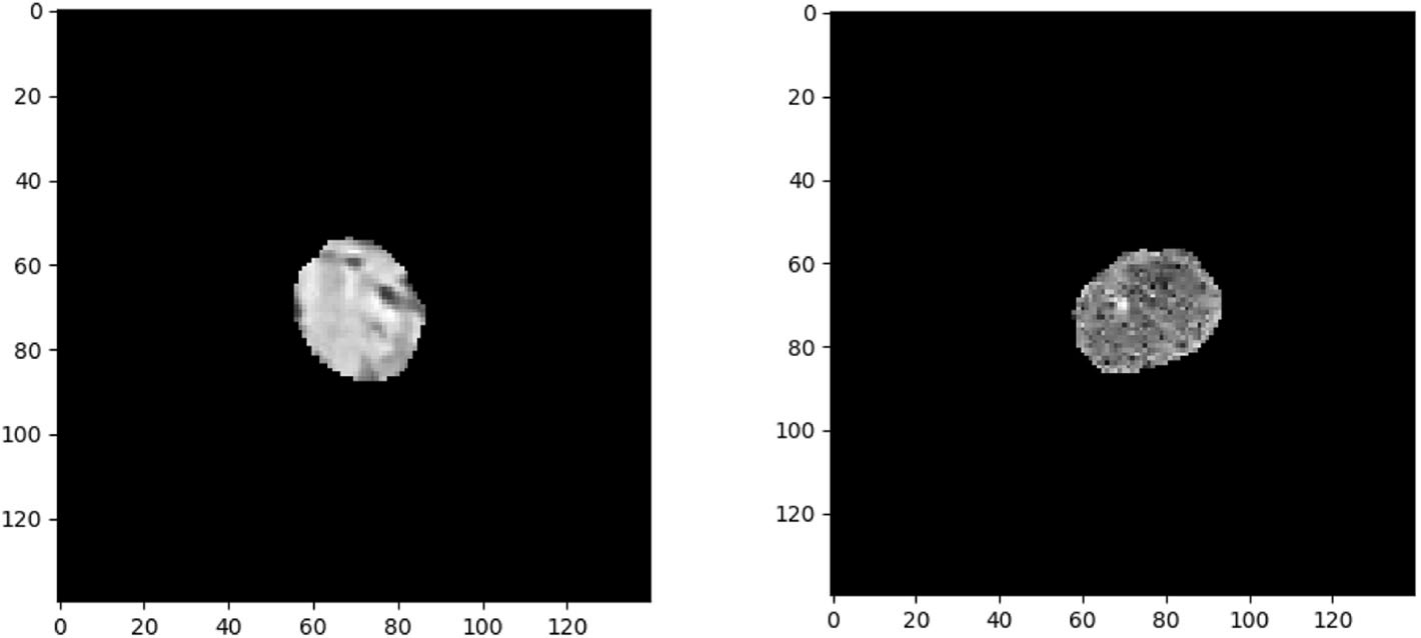
Example of the input CT images. Left: NSCLC tumor from Cohort 1. Right: PDAC tumor from Cohort 2

A CNN architecture with six-layered convolutions (CNN-Survival) was trained as shown in Fig. 1. Input images have dimensions of 140 × 140 × 1 (grayscale), which contain the CT images within the manual contours of the tumors (example shown in Fig. 2). All pixels outside of the contoured region were 0 in the 0 to 255 grayscales. All convolutional layers have kernel size of 3 × 3 with 32 filters following by Batch Normalization layers (BN). The first Max Pool layer has pool size of 2 × 2, and the latter two Max Pool layers have pool size of 3 × 3. Through the Max Pool layers, number of trainable parameters was significantly reduced. To avoid overfitting with this small sample size, dropout layers were added after every two convolutional layers with dropout rate at 0.5. Finally, passing through the flatten and dense layer, images were converted into 19 features and finally, survival probabilities for a given time t were calculated.

### Loss function

To better fit the distribution of survival data, a modified loss function, proposed by Gensheimer et al. [11], was applied to the CNN architecture (Eq. 1).

1 $$ loss=-{\sum}_{i=1}^{d_j}\ln \left({h}_j^i\right)-{\sum}_{i={d}_j+1}^{r_j}\ln \left(1-{h}_j^i\right) $$

In Equation 1, ${h}_j^i$ is the hazard probability for individual i during time interval j. *r* stands for individuals “in view” during the interval j (i.e., survived in this period) and *d* means a patient suffered a failure (e.g., death) during this interval [11]. As it can be seen from Equation 1, the left part penalizes if the model gave low hazard for failure (e.g., death), while the right part penalizes if the model gave high hazard for a survived case. The overall loss function is the sum of the losses for each time interval [11].

### Training process and transfer learning of CNN-survival

**Fig. 3:**
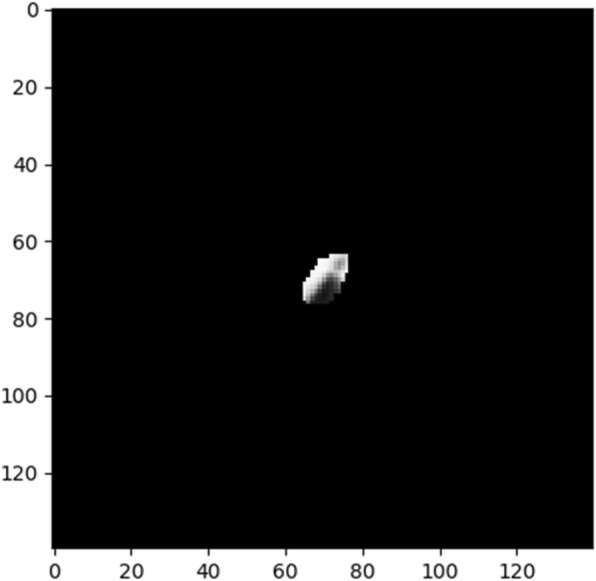
Example of small ROI in Cohort 1

Training a CNN-based survival model needs to finetune a large number of parameters. Given this CNN architecture, there were 73,587 trainable parameters. As such, the larger dataset, cohort 1, was used to pretrain the network. In Cohort 1, 422 patients had 5479 slices containing manually contoured tumor regions. However, the region of interest (ROI) on some of the slices were too small (e.g., less than 250 pixels) to be fed as input to the CNN (shown in Fig. 3). To mitigate this, we ranked slices using their ROI size and pixel intensity and picked the top 2500 slices. This ensured the minimum ROI size of 250 pixels.

These 2500 slices were fed into the proposed CNN model without augmentation. After training the initial model at learning rate 0.0001 for 50 epochs, all the weights in the pretrained model were frozen except for the final dense layer. Next, 68 patients of Cohort 2 were used to finetune the dense layer (containing 627 parameters) for 20 epochs with learning rate of 0.0001 without augmentation. The finetuning was necessary since Cohort 1 and Cohort 2 have CT images from two different types of cancers (lung and pancreas cancer, respectively) with different survival patterns. After 20 epochs of finetuning in Cohort 2, the final model was tested in Cohort 3. The prognosis performance was measured by two metrics: concordance index (CI) [36] and index of prediction accuracy (IPA) [37]. CI is calculated using Equation 2.

2 $$ \mathrm{c}=\frac{1}{\left|\mathcal{E}\right|}\;{\sum}_{{\mathrm{T}}_{\mathrm{i}}\kern0.24em \mathrm{uncensored}}{\sum}_{T_j>{T}_i}{\mathbf{1}}_{\mathbf{f}\left({\mathbf{x}}_{\mathbf{i}}\right)<\mathbf{f}\left({\mathbf{x}}_{\mathbf{j}}\right)} $$

where the indicator function 1_*a* < *b*_ = 1 if a < b, and 0 otherwise. *T*_*i*_ is the survival time for subject i. | $\mathcal{E}$ | is the number of edges in the order graph. f(*x*_*i*_) is the predicted survival time for subject i by model f. Under this formula, concordance index (CI) is the probability of concordance between the predicted and the observed survival [36]. IPA is a recently proposed performance measure for binary and time to event outcomes accounting for both discrimination and calibration, and can identify harmful models as well [37]. IPA of 100% indicates a perfect model, and harmful models will have IPA < 0 [37].

### Radiomics and CNN-survival features

In order to systematically compare the performance of CNN-Survival with CPH models and rule out the confounding variable, we built two additional models using CPH. The first model (Model 1: Radiomics features + LASSO-CPH) is a traditional radiomics-based CPH model, which used 1428 2D radiomics features extracted from the manually contoured regions using PyRadiomics library [2] (version 2.0). A LASSO-CPH [38] feature reduction method was used to find prognostic radiomic features in the training cohort (Cohort 2), which were then tested in the test cohort (Cohort 3). The second model (Model 2: Transfer learning features + LASSO-CPH) was trained using the 19 transfer learning features extracted from the last dense layer of the CNN-Survival model. Similar to Model 1, a LASSO-CPH method was used to select prognostic features in Cohort 2 and test them in Cohort 3. Under this setting, Model 1 and Model 2 had the same type of survival function (LASSO-CPH), and hence, the differences in the input data would explain the differences in performance. On the other hand, Model 2 had the same input data as our proposed CNN-Survival (Model 3) as they both used features from the dense layer. Given that, the performance disparities of Model 2 and Model 3 can be explained by the different survival functions where Model 2 uses LASSO-CPH and instead, Model 3 uses the modified loss function to generate survival probabilities for a given time. The performance of all three models was validated in Cohort 3 (test set) at 18 months by concordance index (CI) and index of prediction accuracy (IPA) using R software (version 3.5.3), Survival, Survcomp, and riskRegression library [39–41].

## Results

In the traditional radiomics with LASSO-CPH approach (Model 1), an optimal CPH model was trained in Cohort 2 using four features (“gradient_gldm_SmallDependenceEmphasis”, “gradient_glszm_SmallAreaEmphasis”, “original_glszm_LargeAreaLowGrayLevelEmphasis”, and “wavelet. HLH_glszm_HighGrayLevelZoneEmphasis”). This model was tested in cohort 3 for validation with CI and IPA at 0.491 and − 3.80%, respectively. Similarly, another LASSO-CPH model (Model 2) was trained using transfer learning features extracted from Cohort 2. Using three features selected by LASSO-CPH, this model yielded CI and IPA of 0.603 and 4.40%, respectively when validated in Cohort 3. In contrast, the proposed CNN-Survival model (Model 3) achieved CI and IPA of 0.651 and 11.81%, respectively, in Cohort 3, outperforming the previous two CPH-based methods. Table 1 lists the results (IPA and CI) for all three survival models.

**Table 1:** Results (IPA and CI) of three survival models for resectable PDAC

	IPA in Cohort 3 (test set)	CI in Cohort 3 (test set)
Model 1: Radiomics features + LASSO-CPH	−3.80%	0.491
Model 2: Transfer learning features + LASSO-CPH	4.40%	0.603
Model 3: Proposed CNN-Survival	11.81%	0.651

**Fig. 4:**
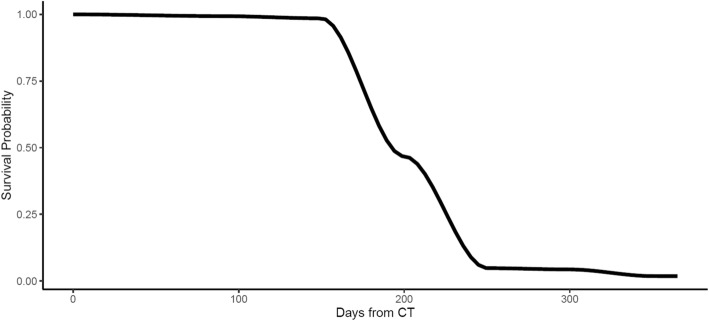
Survival probability curve generated by the proposed CNN-Survival model for a patient deceased 316 days after surgery

**Fig. 5:**
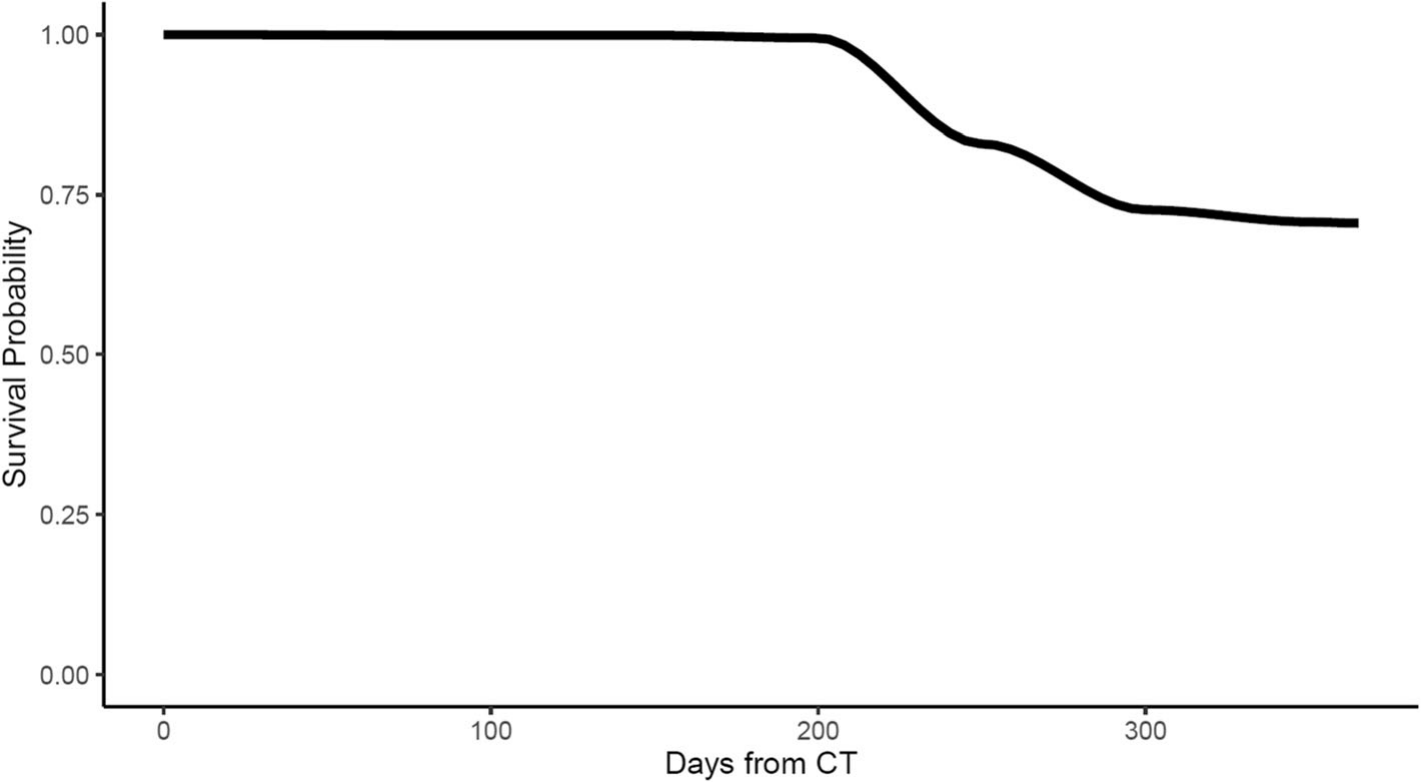
Survival probability curve generated by the proposed CNN-Survival for a patient survived more than one year after surgery

As discussed above, CNN-Survival could depict the survival probability of a patient at a given time. The survival probabilities curves of two patients (one survived versus one deceased) in the test cohort are shown in Fig. 4 and Fig. 5.

For the patient deceased within 1 year after surgery, the survival probability dropped significantly, while for the survived patient, the survival probability stays above 0.5.

## Discussion

Using the proposed CNN-Survival model, the prognosis performance was improved, elevating IPA from − 3.80 to 11.81%, and CI from 0.491 to 0.651 compared to a traditional radiomic-based CPH model (Model 1). Even when transfer learning features were used to build a CPH model (Model 2), the proposed CNN-Survival model was still superior (IPA: 11.81% vs 4.40%, CI: 0.651 vs. 0.603). These comparisons illustrate that transfer learning features outperform radiomics features (Model 2 vs. Model 1) and the proposed CNN-Survival model using a modified loss function outperforms both CPH-based models (Model 3 vs. Model 1 and 2). Deep learning networks provide flexibility in modifying the dimension of feature space and loss function, enabling us to extract disease-specific features and build more precise models. Using a CNN-based survival model, we showed that, with the help of transfer learning, deep learning architectures can outperform the traditional pipeline in a typical small sample size setting when modeling the survival for resectable PDAC patients. The proposed transfer learning-based CNN-Survival model has significant potential to enable researchers to pretrain a model using images from common cancers with larger datasets and transfer this model to target rare cancers. Transfer learning-based CNN-Survival model mitigates the needs for large sample size, allowing the survival model to be applied to a wide range of cancer sites.

The proposed CNN-Survival model provides better prognostic performance compared to the traditional radiomics analytic pipeline (IPA 11.81% versus − 3.80%). Although there was no prior publications reporting IPA for PDAC biomarkers, the IPA of our proposed CNN-Survival is comparable to the typical IPA for other survival models [37]. From the feature extraction perspective, parameters in a CNN can be updated during backpropagation, allowing to extract a large number of features that are associated with the target outcome. For feature analysis, the CNN-Survival model avoids the multiple testing, which is a significant issue in the conventional radiomics analytic pipeline. Finally, with the modified loss function, CNN-Survival model does not rely on the linear assumption, making it suitable for more real-world scenarios. These advantages contributed to the improved performance of the proposed model. Compared to transfer learning features-based CPH model (Model 2), which used the same feature sued by CNN-Survival model (Model 3), the proposed CNN-Survival had higher IPA (11.89% versus 4.40%). Given that these two models had the same data input, this result indicates that the loss function in CNN-Survival model outperforms the traditional linear CPH which is commonly used in radiomic studies.

In this research, due to the small sample size in PDAC cohorts, the proposed CNN-Survival model was not optimal. We used CT images from 68 patients to finetune the pretrained CNN-Survival model and tested in another 30 patients of an independent cohort. Although through transfer learning, most of the parameters were trained using the pretrained cohort, there were still 627 parameters in the dense layer needed to be modified through finetuning. Thus, if a larger dataset was available for finetuning, performance may be further improved. Additionally, the pretrained dataset are CT images from Non-Small Cell Lung Cancer (NSCLC) patients. Although it is the largest open source dataset we could find, NSCLC has different biological traits and survival patterns compared to PDAC. In future research, using a similar pretrained domain and a larger finetuning cohort, further improvement may be achieved. A proper validation of the proposed model is required through clinical validation, which is beyond the scope of this work.

In this study, using CT images from three independent cohorts, we validated the proposed CNN-Survival model with the modified loss function proposed by Gensheimer et al. [11]. We showed that the proposed CNN-Survival model outperformed and avoided the limitations of the conventional radiomics-based CPH model in a real-world small sample size setting. Further validation of this loss function can be performed for other types of diseases through transfer learning. The proposed CNN-Survival model has the potential to be a standardized survival model in quantitative medical imaging research field.

## Conclusions

The proposed CNN-based survival model outperforms traditional CPH-based radiomics and transfer learning pipelines in PDAC prognosis. This approach offers a better fit for survival patterns based on CT images and overcomes the limitations of conventional survival models.

## Data Availability

The datasets of Cohort 2 and Cohort 3 analyzed during the current study are available from the corresponding author on reasonable request pending the approval of the institution(s) and trial/study investigators who contributed to the dataset.

## Abbreviations

ANN: Artificial Neural Networks
BN: Batch Normalization
CI: Concordance Index
CNN: Convolutional Neural Network
CPH: Cox Proportion Hazard
CT: Computerized Tomography
NSCLC: Non-Small Cell Lung Cancer
PDAC: Pancreatic Ductal Adenocarcinoma
ROI: Region of Interest
SVM: Support Vector Machine
